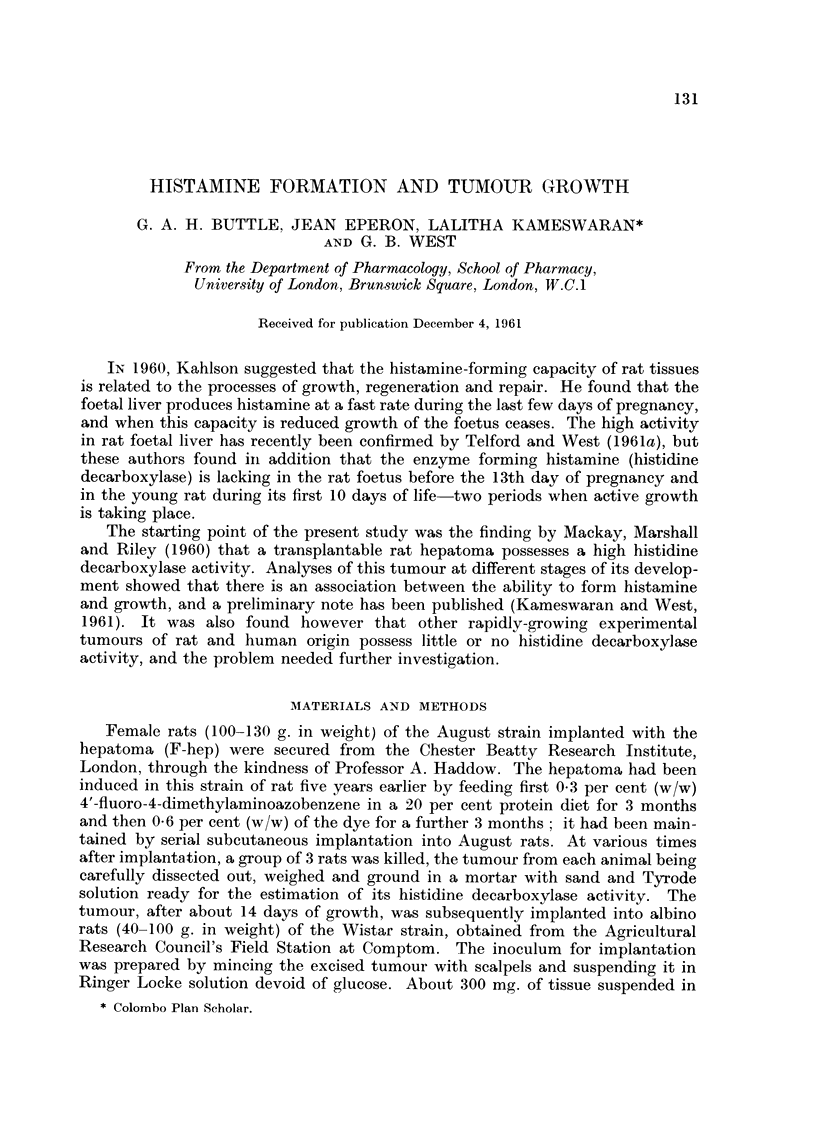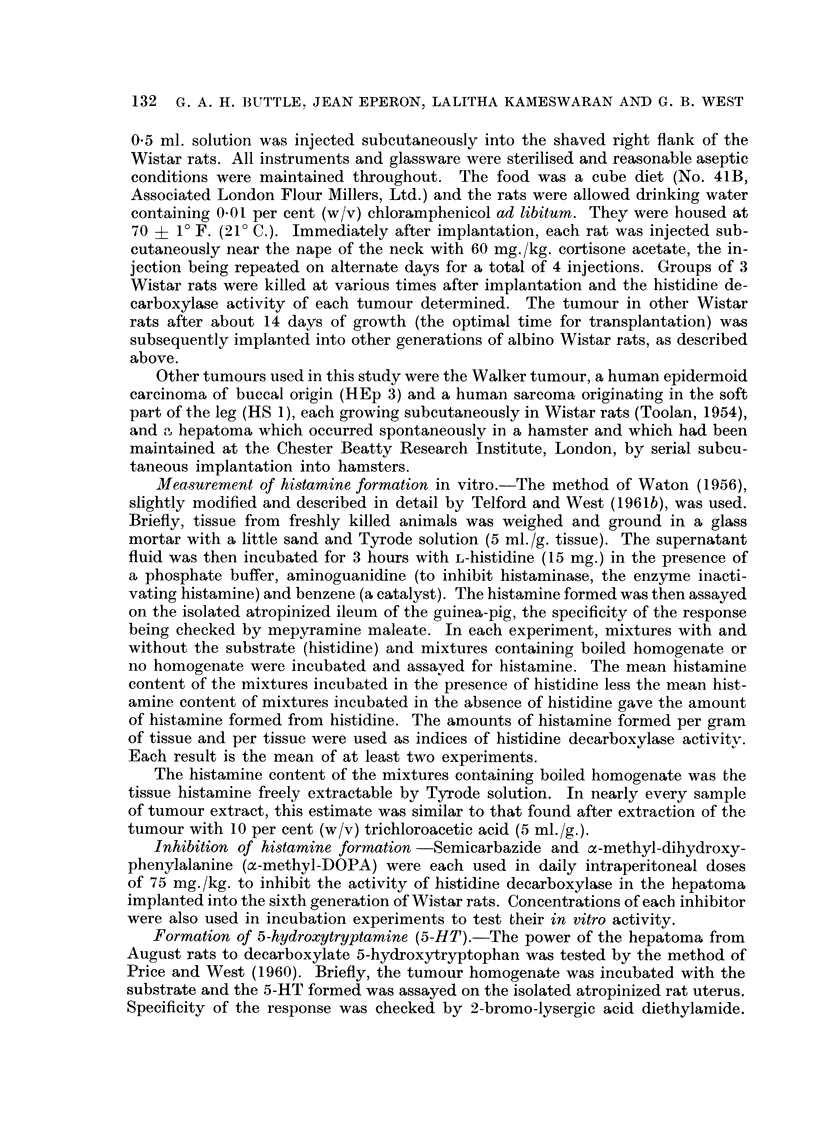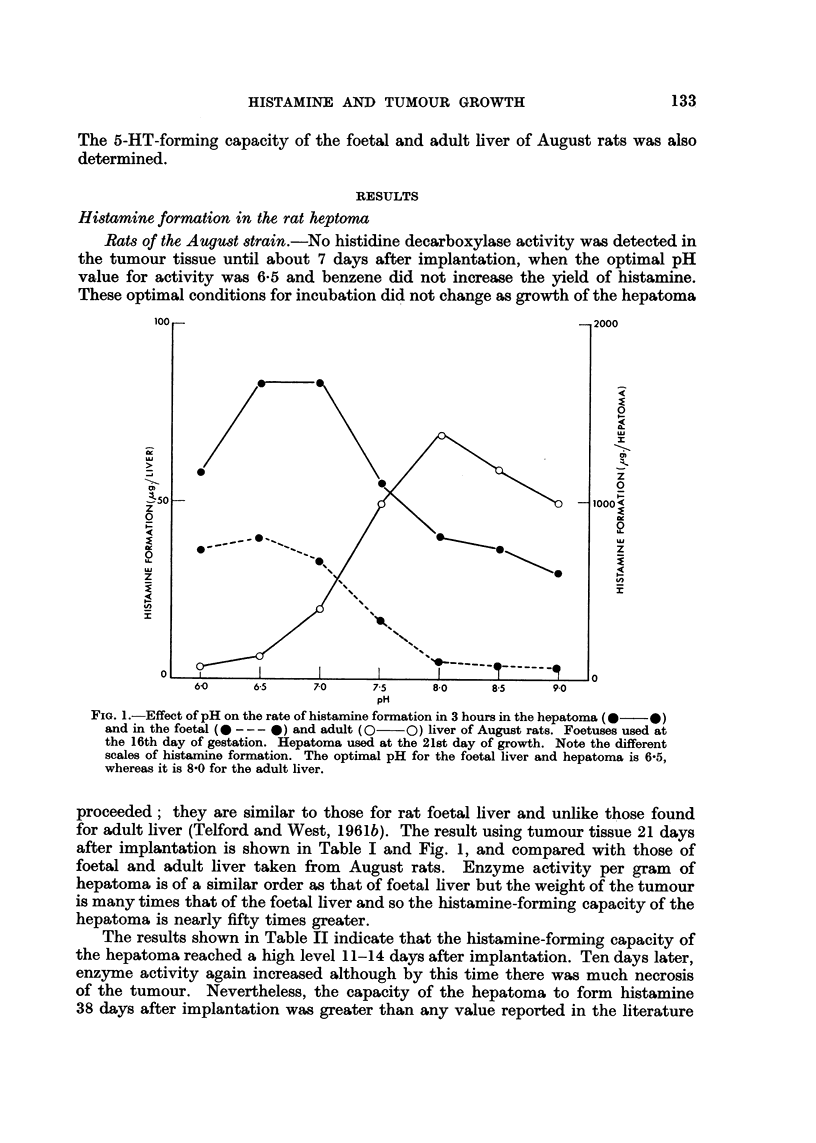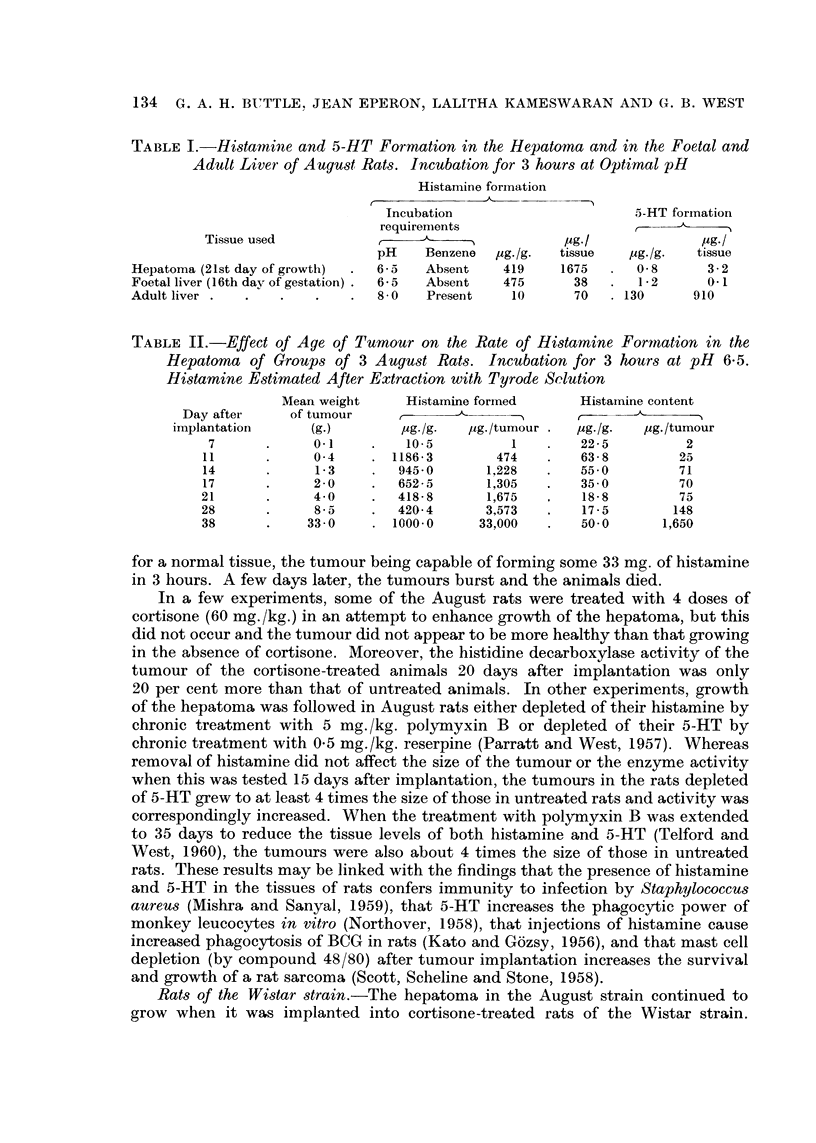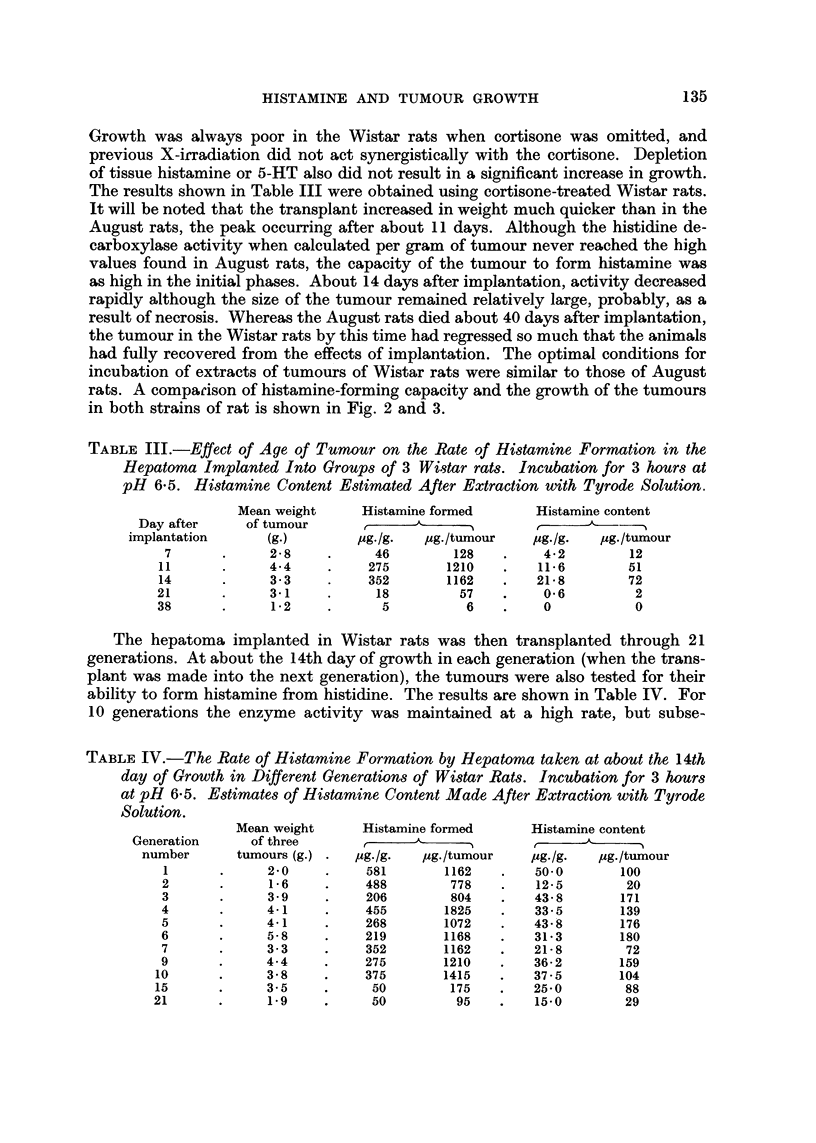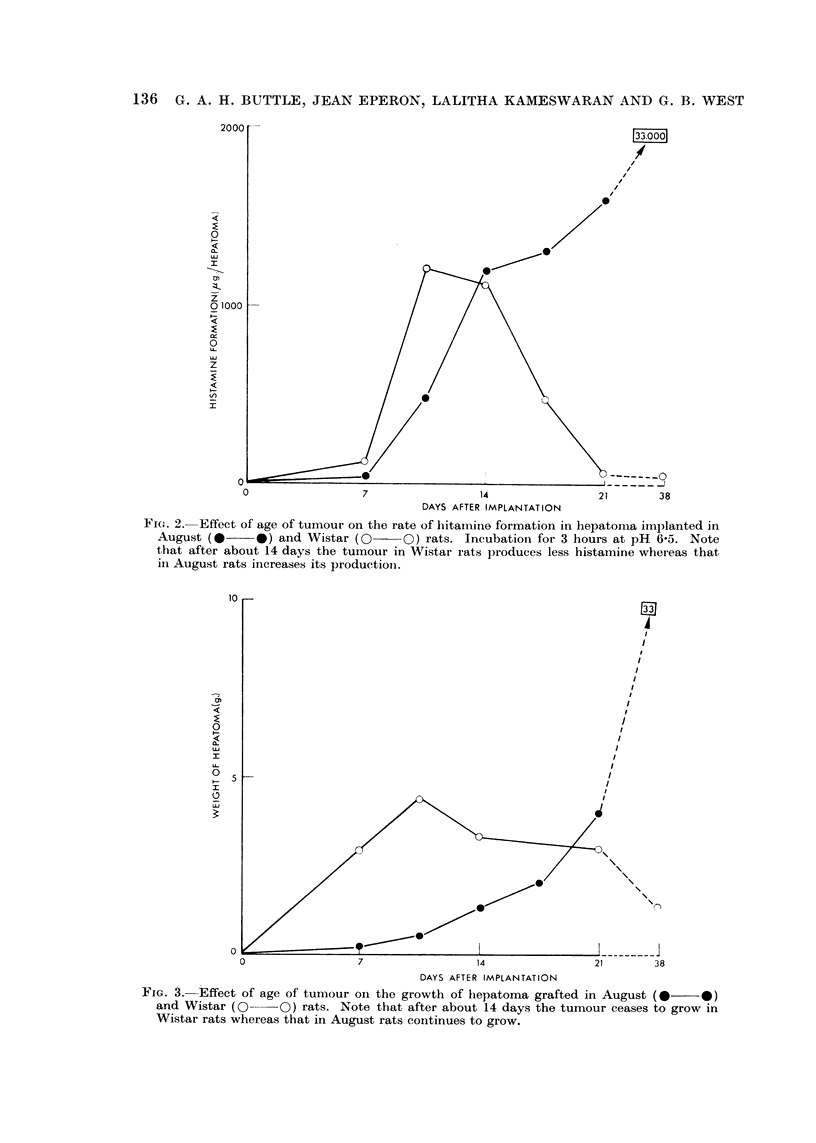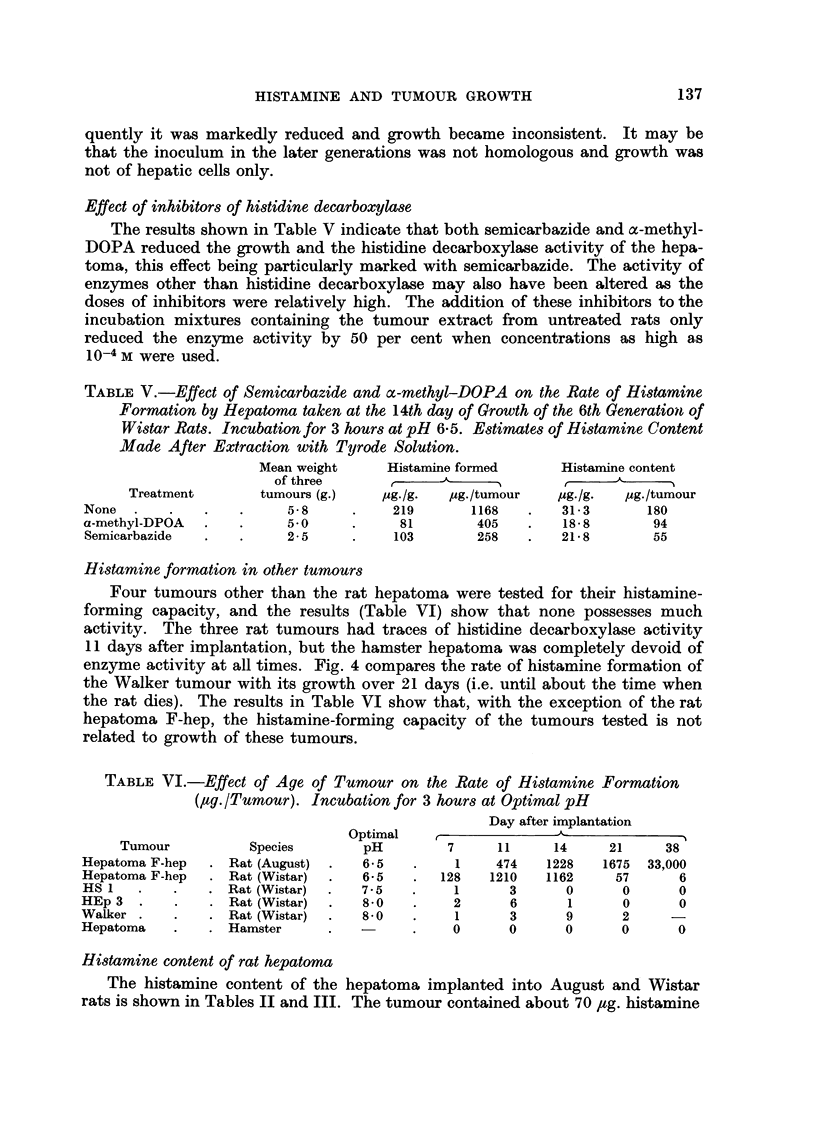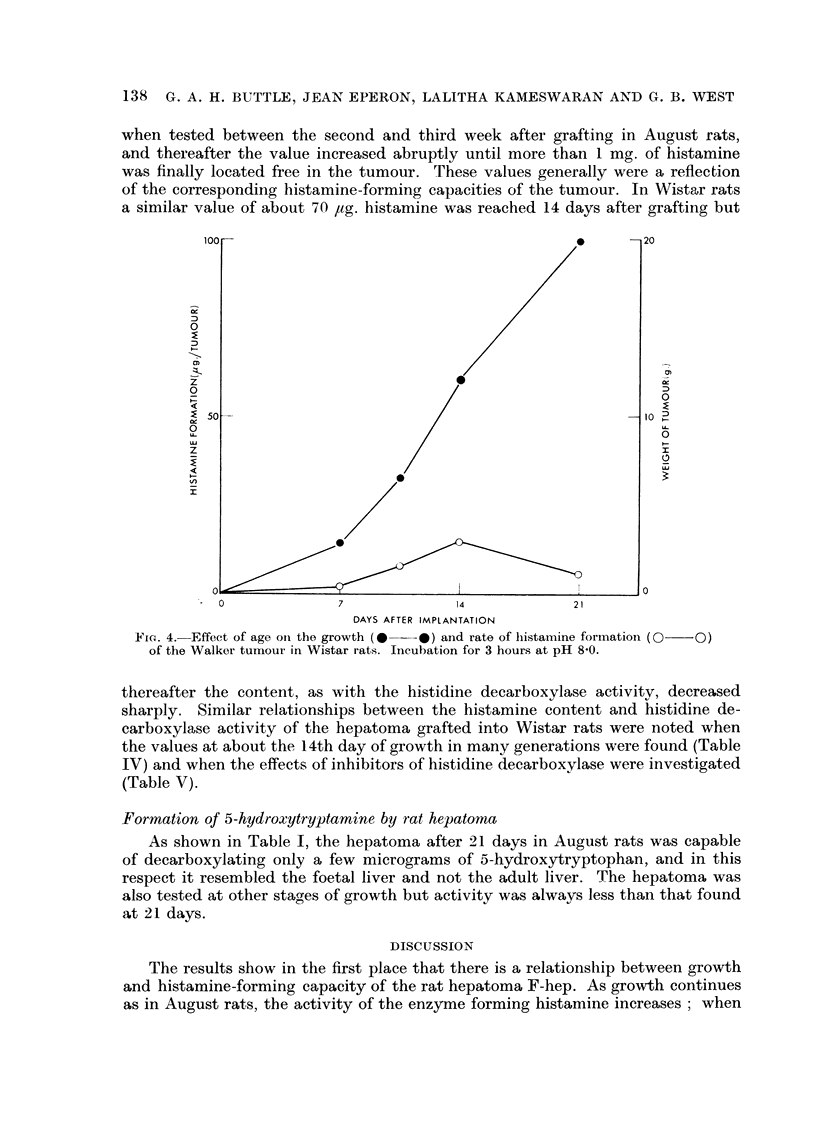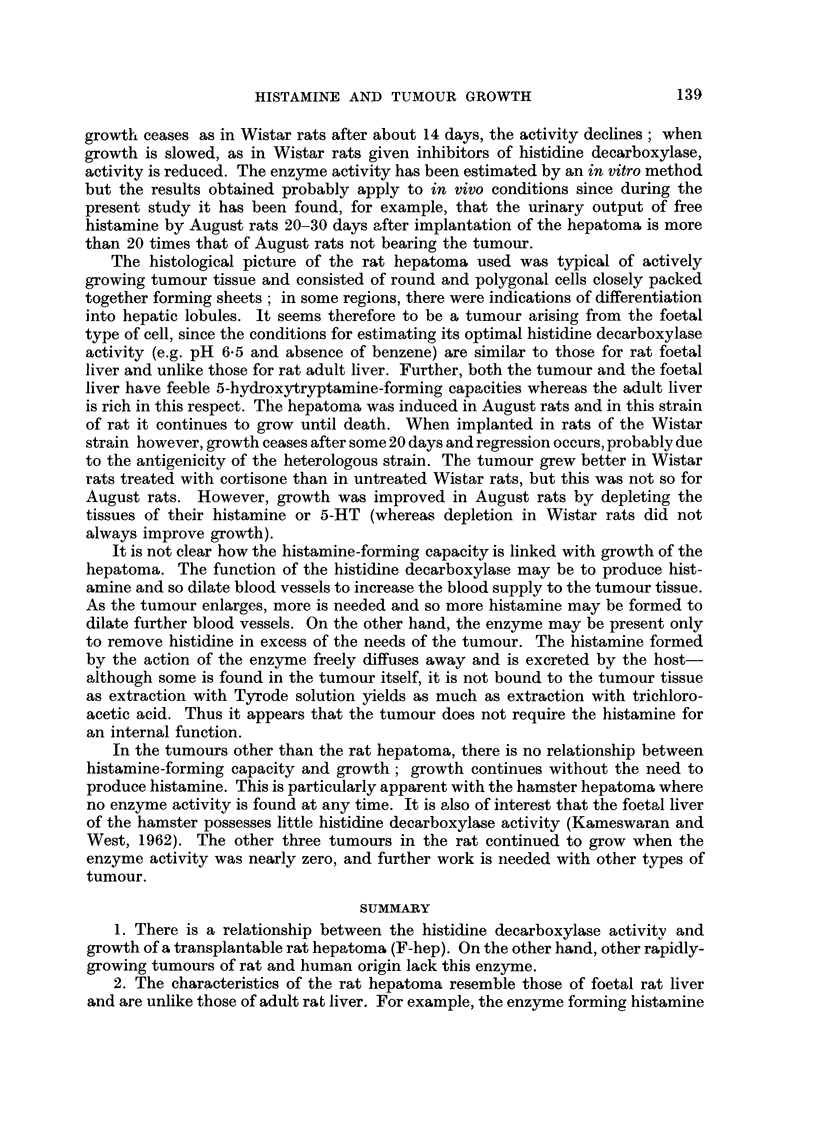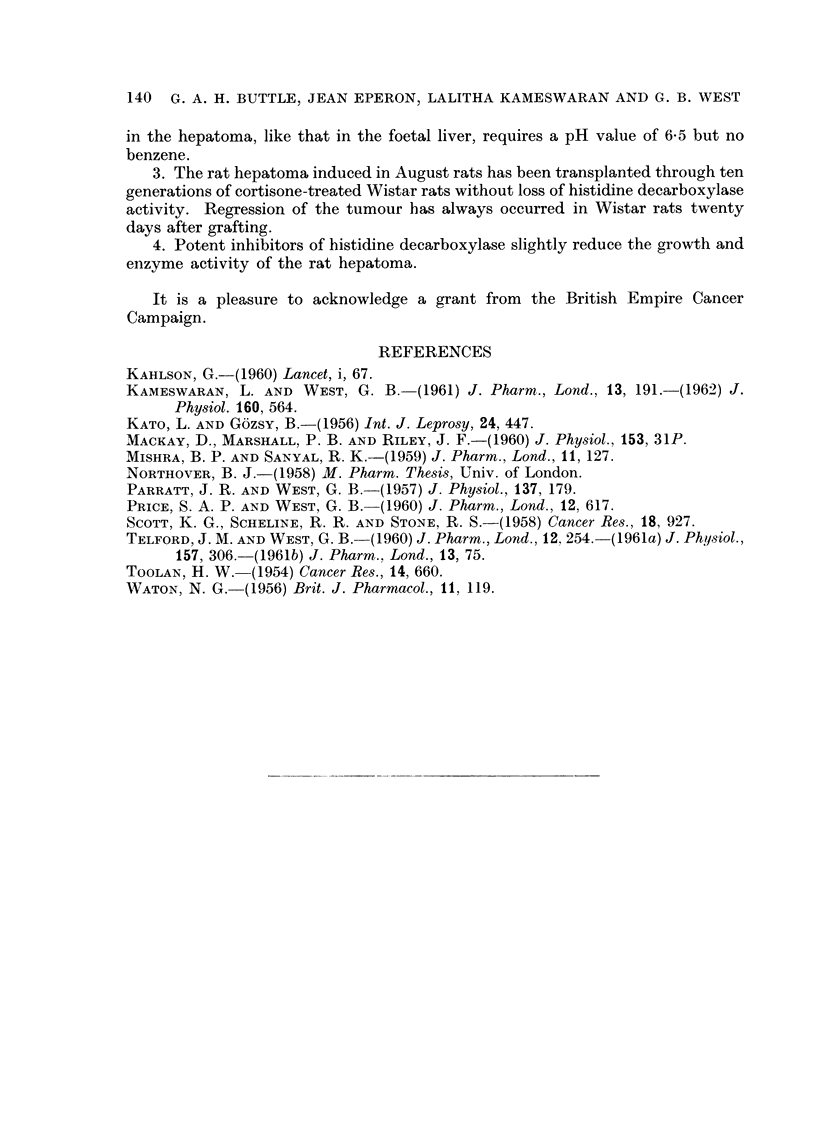# Histamine Formation and Tumour Growth

**DOI:** 10.1038/bjc.1962.13

**Published:** 1962-03

**Authors:** G. A. H. Buttle, Jean Eperon, Lalitha Kameswaran, G. B. West


### HISTAMINE FORMATION AND TUMOUR GROWTH

G. A. H. BUTTLE, JEAN EPERON, LALITHA KAMESWARAN*
AND G. B. WEST

*From the Department of Pharmacology, School of Pharmacy, University of London, Brunswick Square, London, W.C.1*

Received for publication December 4, 1961

IN 1960, Kahlson suggested that the histamine-forming capacity of rat tissues is related to the processes of growth, regeneration and repair. He found that the foetal liver produces histamine at a fast rate during the last few days of pregnancy, and when this capacity is reduced growth of the foetus ceases. The high activity in rat foetal liver has recently been confirmed by Telford and West (1961*a*), but these authors found in addition that the enzyme forming histamine (histidine decarboxylase) is lacking in the rat foetus before the 13th day of pregnancy and in the young rat during its first 10 days of life—two periods when active growth is taking place.

The starting point of the present study was the finding by Mackay, Marshall and Riley (1960) that a transplantable rat hepatoma possesses a high histidine decarboxylase activity. Analyses of this tumour at different stages of its development showed that there is an association between the ability to form histamine and growth, and a preliminary note has been published (Kameswaran and West, 1961). It was also found however that other rapidly-growing experimental tumours of rat and human origin possess little or no histidine decarboxylase activity, and the problem needed further investigation.

### MATERIALS AND METHODS

Female rats (100–130 g. in weight) of the August strain implanted with the hepatoma (F-hep) were secured from the Chester Beatty Research Institute, London, through the kindness of Professor A. Haddow. The hepatoma had been induced in this strain of rat five years earlier by feeding first 0·3 per cent (w/w) 4′-fluoro-4-dimethylaminoazobenzene in a 20 per cent protein diet for 3 months and then 0·6 per cent (w/w) of the dye for a further 3 months ; it had been maintained by serial subcutaneous implantation into August rats. At various times after implantation, a group of 3 rats was killed, the tumour from each animal being carefully dissected out, weighed and ground in a mortar with sand and Tyrode solution ready for the estimation of its histidine decarboxylase activity. The tumour, after about 14 days of growth, was subsequently implanted into albino rats (40–100 g. in weight) of the Wistar strain, obtained from the Agricultural Research Council's Field Station at Comptom. The inoculum for implantation was prepared by mincing the excised tumour with scalpels and suspending it in Ringer Locke solution devoid of glucose. About 300 mg. of tissue suspended in

* Colombo Plan Scholar.

0·5 ml. solution was injected subcutaneously into the shaved right flank of the Wistar rats. All instruments and glassware were sterilised and reasonable aseptic conditions were maintained throughout. The food was a cube diet (No. 41B, Associated London Flour Millers, Ltd.) and the rats were allowed drinking water containing 0·01 per cent (w/v) chloramphenicol *ad libitum*. They were housed at 70 $\pm$ 1° F. (21° C.). Immediately after implantation, each rat was injected subcutaneously near the nape of the neck with 60 mg./kg. cortisone acetate, the injection being repeated on alternate days for a total of 4 injections. Groups of 3 Wistar rats were killed at various times after implantation and the histidine decarboxylase activity of each tumour determined. The tumour in other Wistar rats after about 14 days of growth (the optimal time for transplantation) was subsequently implanted into other generations of albino Wistar rats, as described above.

Other tumours used in this study were the Walker tumour, a human epidermoid carcinoma of buccal origin (HEp 3) and a human sarcoma originating in the soft part of the leg (HS 1), each growing subcutaneously in Wistar rats (Toolan, 1954), and a hepatoma which occurred spontaneously in a hamster and which had been maintained at the Chester Beatty Research Institute, London, by serial subcutaneous implantation into hamsters.

*Measurement of histamine formation* in vitro.—The method of Waton (1956), slightly modified and described in detail by Telford and West (1961*b*), was used. Briefly, tissue from freshly killed animals was weighed and ground in a glass mortar with a little sand and Tyrode solution (5 ml./g. tissue). The supernatant fluid was then incubated for 3 hours with L-histidine (15 mg.) in the presence of a phosphate buffer, aminoguanidine (to inhibit histaminase, the enzyme inactivating histamine) and benzene (a catalyst). The histamine formed was then assayed on the isolated atropinized ileum of the guinea-pig, the specificity of the response being checked by mepyramine maleate. In each experiment, mixtures with and without the substrate (histidine) and mixtures containing boiled homogenate or no homogenate were incubated and assayed for histamine. The mean histamine content of the mixtures incubated in the presence of histidine less the mean histamine content of mixtures incubated in the absence of histidine gave the amount of histamine formed from histidine. The amounts of histamine formed per gram of tissue and per tissue were used as indices of histidine decarboxylase activity. Each result is the mean of at least two experiments.

The histamine content of the mixtures containing boiled homogenate was the tissue histamine freely extractable by Tyrode solution. In nearly every sample of tumour extract, this estimate was similar to that found after extraction of the tumour with 10 per cent (w/v) trichloroacetic acid (5 ml./g.).

*Inhibition of histamine formation*.—Semicarbazide and $\alpha$-methyl-dihydroxyphenylalanine ($\alpha$-methyl-DOPA) were each used in daily intraperitoneal doses of 75 mg./kg. to inhibit the activity of histidine decarboxylase in the hepatoma implanted into the sixth generation of Wistar rats. Concentrations of each inhibitor were also used in incubation experiments to test their *in vitro* activity.

*Formation of 5-hydroxytryptamine* (*5-HT*).—The power of the hepatoma from August rats to decarboxylate 5-hydroxytryptophan was tested by the method of Price and West (1960). Briefly, the tumour homogenate was incubated with the substrate and the 5-HT formed was assayed on the isolated atropinized rat uterus. Specificity of the response was checked by 2-bromo-lysergic acid diethylamide.

The 5-HT-forming capacity of the foetal and adult liver of August rats was also determined.

### RESULTS

### *Histamine formation in the rat heptoma*

*Rats of the August strain.*—No histidine decarboxylase activity was detected in the tumour tissue until about 7 days after implantation, when the optimal pH value for activity was 6·5 and benzene did not increase the yield of histamine. These optimal conditions for incubation did not change as growth of the hepatoma

Fig. 1.—Effect of pH on the rate of histamine formation in 3 hours in the hepatoma (●——●) and in the foetal (● – – – ●) and adult (○——○) liver of August rats. Foetuses used at the 16th day of gestation. Hepatoma used at the 21st day of growth. Note the different scales of histamine formation. The optimal pH for the foetal liver and hepatoma is 6·5, whereas it is 8·0 for the adult liver.

proceeded ; they are similar to those for rat foetal liver and unlike those found for adult liver (Telford and West, 1961*b*). The result using tumour tissue 21 days after implantation is shown in Table I and Fig. 1, and compared with those of foetal and adult liver taken from August rats. Enzyme activity per gram of hepatoma is of a similar order as that of foetal liver but the weight of the tumour is many times that of the foetal liver and so the histamine-forming capacity of the hepatoma is nearly fifty times greater.

The results shown in Table II indicate that the histamine-forming capacity of the hepatoma reached a high level 11–14 days after implantation. Ten days later, enzyme activity again increased although by this time there was much necrosis of the tumour. Nevertheless, the capacity of the hepatoma to form histamine 38 days after implantation was greater than any value reported in the literature

TABLE I.—*Histamine and 5-HT Formation in the Hepatoma and in the Foetal and Adult Liver of August Rats. Incubation for 3 hours at Optimal pH*

| Tissue used | Histamine formation: Incubation requirements: pH | Benzene | μg./g. | μg./tissue | 5-HT formation: μg./g. | μg./tissue |
|---|---|---|---|---|---|---|
| Hepatoma (21st day of growth) | 6·5 | Absent | 419 | 1675 | 0·8 | 3·2 |
| Foetal liver (16th day of gestation) | 6·5 | Absent | 475 | 38 | 1·2 | 0·1 |
| Adult liver | 8·0 | Present | 10 | 70 | 130 | 910 |

TABLE II.—*Effect of Age of Tumour on the Rate of Histamine Formation in the Hepatoma of Groups of 3 August Rats. Incubation for 3 hours at pH 6·5. Histamine Estimated After Extraction with Tyrode Solution*

| Day after implantation | Mean weight of tumour (g.) | Histamine formed: μg./g. | μg./tumour | Histamine content: μg./g. | μg./tumour |
|---|---|---|---|---|---|
| 7 | 0·1 | 10·5 | 1 | 22·5 | 2 |
| 11 | 0·4 | 1186·3 | 474 | 63·8 | 25 |
| 14 | 1·3 | 945·0 | 1,228 | 55·0 | 71 |
| 17 | 2·0 | 652·5 | 1,305 | 35·0 | 70 |
| 21 | 4·0 | 418·8 | 1,675 | 18·8 | 75 |
| 28 | 8·5 | 420·4 | 3,573 | 17·5 | 148 |
| 38 | 33·0 | 1000·0 | 33,000 | 50·0 | 1,650 |

for a normal tissue, the tumour being capable of forming some 33 mg. of histamine in 3 hours. A few days later, the tumours burst and the animals died.

In a few experiments, some of the August rats were treated with 4 doses of cortisone (60 mg./kg.) in an attempt to enhance growth of the hepatoma, but this did not occur and the tumour did not appear to be more healthy than that growing in the absence of cortisone. Moreover, the histidine decarboxylase activity of the tumour of the cortisone-treated animals 20 days after implantation was only 20 per cent more than that of untreated animals. In other experiments, growth of the hepatoma was followed in August rats either depleted of their histamine by chronic treatment with 5 mg./kg. polymyxin B or depleted of their 5-HT by chronic treatment with 0·5 mg./kg. reserpine (Parratt and West, 1957). Whereas removal of histamine did not affect the size of the tumour or the enzyme activity when this was tested 15 days after implantation, the tumours in the rats depleted of 5-HT grew to at least 4 times the size of those in untreated rats and activity was correspondingly increased. When the treatment with polymyxin B was extended to 35 days to reduce the tissue levels of both histamine and 5-HT (Telford and West, 1960), the tumours were also about 4 times the size of those in untreated rats. These results may be linked with the findings that the presence of histamine and 5-HT in the tissues of rats confers immunity to infection by *Staphylococcus aureus* (Mishra and Sanyal, 1959), that 5-HT increases the phagocytic power of monkey leucocytes *in vitro* (Northover, 1958), that injections of histamine cause increased phagocytosis of BCG in rats (Kato and Gözsy, 1956), and that mast cell depletion (by compound 48/80) after tumour implantation increases the survival and growth of a rat sarcoma (Scott, Scheline and Stone, 1958).

*Rats of the Wistar strain.*—The hepatoma in the August strain continued to grow when it was implanted into cortisone-treated rats of the Wistar strain.

Growth was always poor in the Wistar rats when cortisone was omitted, and previous X-irradiation did not act synergistically with the cortisone. Depletion of tissue histamine or 5-HT also did not result in a significant increase in growth. The results shown in Table III were obtained using cortisone-treated Wistar rats. It will be noted that the transplant increased in weight much quicker than in the August rats, the peak occurring after about 11 days. Although the histidine decarboxylase activity when calculated per gram of tumour never reached the high values found in August rats, the capacity of the tumour to form histamine was as high in the initial phases. About 14 days after implantation, activity decreased rapidly although the size of the tumour remained relatively large, probably, as a result of necrosis. Whereas the August rats died about 40 days after implantation, the tumour in the Wistar rats by this time had regressed so much that the animals had fully recovered from the effects of implantation. The optimal conditions for incubation of extracts of tumours of Wistar rats were similar to those of August rats. A comparison of histamine-forming capacity and the growth of the tumours in both strains of rat is shown in Fig. 2 and 3.

TABLE III.—*Effect of Age of Tumour on the Rate of Histamine Formation in the Hepatoma Implanted Into Groups of* 3 *Wistar rats. Incubation for* 3 *hours at pH* 6·5. *Histamine Content Estimated After Extraction with Tyrode Solution.*

| Day after implantation | Mean weight of tumour (g.) | Histamine formed | | Histamine content | |
|---|---|---|---|---|---|
| | | μg./g. | μg./tumour | μg./g. | μg./tumour |
| 7 | 2·8 | 46 | 128 | 4·2 | 12 |
| 11 | 4·4 | 275 | 1210 | 11·6 | 51 |
| 14 | 3·3 | 352 | 1162 | 21·8 | 72 |
| 21 | 3·1 | 18 | 57 | 0·6 | 2 |
| 38 | 1·2 | 5 | 6 | 0 | 0 |

The hepatoma implanted in Wistar rats was then transplanted through 21 generations. At about the 14th day of growth in each generation (when the transplant was made into the next generation), the tumours were also tested for their ability to form histamine from histidine. The results are shown in Table IV. For 10 generations the enzyme activity was maintained at a high rate, but subse-

TABLE IV.—*The Rate of Histamine Formation by Hepatoma taken at about the* 14*th day of Growth in Different Generations of Wistar Rats. Incubation for* 3 *hours at pH* 6·5. *Estimates of Histamine Content Made After Extraction with Tyrode Solution.*

| Generation number | Mean weight of three tumours (g.) | Histamine formed | | Histamine content | |
|---|---|---|---|---|---|
| | | μg./g. | μg./tumour | μg./g. | μg./tumour |
| 1 | 2·0 | 581 | 1162 | 50·0 | 100 |
| 2 | 1·6 | 488 | 778 | 12·5 | 20 |
| 3 | 3·9 | 206 | 804 | 43·8 | 171 |
| 4 | 4·1 | 455 | 1825 | 33·5 | 139 |
| 5 | 4·1 | 268 | 1072 | 43·8 | 176 |
| 6 | 5·8 | 219 | 1168 | 31·3 | 180 |
| 7 | 3·3 | 352 | 1162 | 21·8 | 72 |
| 9 | 4·4 | 275 | 1210 | 36·2 | 159 |
| 10 | 3·8 | 375 | 1415 | 37·5 | 104 |
| 15 | 3·5 | 50 | 175 | 25·0 | 88 |
| 21 | 1·9 | 50 | 95 | 15·0 | 29 |

FIG. 2.—Effect of age of tumour on the rate of hitamine formation in hepatoma implanted in August (●——●) and Wistar (○——○) rats. Incubation for 3 hours at pH 6·5. Note that after about 14 days the tumour in Wistar rats produces less histamine whereas that in August rats increases its production.

FIG. 3.—Effect of age of tumour on the growth of hepatoma grafted in August (●——●) and Wistar (○——○) rats. Note that after about 14 days the tumour ceases to grow in Wistar rats whereas that in August rats continues to grow.

quently it was markedly reduced and growth became inconsistent. It may be that the inoculum in the later generations was not homologous and growth was not of hepatic cells only.

*Effect of inhibitors of histidine decarboxylase*

The results shown in Table V indicate that both semicarbazide and α-methyl-DOPA reduced the growth and the histidine decarboxylase activity of the hepatoma, this effect being particularly marked with semicarbazide. The activity of enzymes other than histidine decarboxylase may also have been altered as the doses of inhibitors were relatively high. The addition of these inhibitors to the incubation mixtures containing the tumour extract from untreated rats only reduced the enzyme activity by 50 per cent when concentrations as high as $10^{-4}$ M were used.

TABLE V.—*Effect of Semicarbazide and α-methyl-DOPA on the Rate of Histamine Formation by Hepatoma taken at the 14th day of Growth of the 6th Generation of Wistar Rats. Incubation for 3 hours at pH 6·5. Estimates of Histamine Content Made After Extraction with Tyrode Solution.*

| Treatment | Mean weight of three tumours (g.) | Histamine formed | | Histamine content | |
|---|---|---|---|---|---|
| | | μg./g. | μg./tumour | μg./g. | μg./tumour |
| None | 5·8 | 219 | 1168 | 31·3 | 180 |
| α-methyl-DPOA | 5·0 | 81 | 405 | 18·8 | 94 |
| Semicarbazide | 2·5 | 103 | 258 | 21·8 | 55 |

*Histamine formation in other tumours*

Four tumours other than the rat hepatoma were tested for their histamine-forming capacity, and the results (Table VI) show that none possesses much activity. The three rat tumours had traces of histidine decarboxylase activity 11 days after implantation, but the hamster hepatoma was completely devoid of enzyme activity at all times. Fig. 4 compares the rate of histamine formation of the Walker tumour with its growth over 21 days (i.e. until about the time when the rat dies). The results in Table VI show that, with the exception of the rat hepatoma F-hep, the histamine-forming capacity of the tumours tested is not related to growth of these tumours.

TABLE VI.—*Effect of Age of Tumour on the Rate of Histamine Formation (μg./Tumour). Incubation for 3 hours at Optimal pH*

| Tumour | Species | Optimal pH | Day after implantation | | | | |
|---|---|---|---|---|---|---|---|
| | | | 7 | 11 | 14 | 21 | 38 |
| Hepatoma F-hep | Rat (August) | 6·5 | 1 | 474 | 1228 | 1675 | 33,000 |
| Hepatoma F-hep | Rat (Wistar) | 6·5 | 128 | 1210 | 1162 | 57 | 6 |
| HS 1 | Rat (Wistar) | 7·5 | 1 | 3 | 0 | 0 | 0 |
| HEp 3 | Rat (Wistar) | 8·0 | 2 | 6 | 1 | 0 | 0 |
| Walker | Rat (Wistar) | 8·0 | 1 | 3 | 9 | 2 | — |
| Hepatoma | Hamster | — | 0 | 0 | 0 | 0 | 0 |

*Histamine content of rat hepatoma*

The histamine content of the hepatoma implanted into August and Wistar rats is shown in Tables II and III. The tumour contained about 70 μg. histamine when tested between the second and third week after grafting in August rats, and thereafter the value increased abruptly until more than 1 mg. of histamine was finally located free in the tumour. These values generally were a reflection of the corresponding histamine-forming capacities of the tumour. In Wistar rats a similar value of about 70 $\mu$g. histamine was reached 14 days after grafting but

FIG. 4.—Effect of age on the growth (●——●) and rate of histamine formation (○——○) of the Walker tumour in Wistar rats. Incubation for 3 hours at pH 8·0.

thereafter the content, as with the histidine decarboxylase activity, decreased sharply. Similar relationships between the histamine content and histidine decarboxylase activity of the hepatoma grafted into Wistar rats were noted when the values at about the 14th day of growth in many generations were found (Table IV) and when the effects of inhibitors of histidine decarboxylase were investigated (Table V).

*Formation of 5-hydroxytryptamine by rat hepatoma*

As shown in Table I, the hepatoma after 21 days in August rats was capable of decarboxylating only a few micrograms of 5-hydroxytryptophan, and in this respect it resembled the foetal liver and not the adult liver. The hepatoma was also tested at other stages of growth but activity was always less than that found at 21 days.

### DISCUSSION

The results show in the first place that there is a relationship between growth and histamine-forming capacity of the rat hepatoma F-hep. As growth continues as in August rats, the activity of the enzyme forming histamine increases ; when growth ceases as in Wistar rats after about 14 days, the activity declines ; when growth is slowed, as in Wistar rats given inhibitors of histidine decarboxylase, activity is reduced. The enzyme activity has been estimated by an *in vitro* method but the results obtained probably apply to *in vivo* conditions since during the present study it has been found, for example, that the urinary output of free histamine by August rats 20–30 days after implantation of the hepatoma is more than 20 times that of August rats not bearing the tumour.

The histological picture of the rat hepatoma used was typical of actively growing tumour tissue and consisted of round and polygonal cells closely packed together forming sheets ; in some regions, there were indications of differentiation into hepatic lobules. It seems therefore to be a tumour arising from the foetal type of cell, since the conditions for estimating its optimal histidine decarboxylase activity (e.g. pH 6·5 and absence of benzene) are similar to those for rat foetal liver and unlike those for rat adult liver. Further, both the tumour and the foetal liver have feeble 5-hydroxytryptamine-forming capacities whereas the adult liver is rich in this respect. The hepatoma was induced in August rats and in this strain of rat it continues to grow until death. When implanted in rats of the Wistar strain however, growth ceases after some 20 days and regression occurs, probably due to the antigenicity of the heterologous strain. The tumour grew better in Wistar rats treated with cortisone than in untreated Wistar rats, but this was not so for August rats. However, growth was improved in August rats by depleting the tissues of their histamine or 5-HT (whereas depletion in Wistar rats did not always improve growth).

It is not clear how the histamine-forming capacity is linked with growth of the hepatoma. The function of the histidine decarboxylase may be to produce histamine and so dilate blood vessels to increase the blood supply to the tumour tissue. As the tumour enlarges, more is needed and so more histamine may be formed to dilate further blood vessels. On the other hand, the enzyme may be present only to remove histidine in excess of the needs of the tumour. The histamine formed by the action of the enzyme freely diffuses away and is excreted by the host—although some is found in the tumour itself, it is not bound to the tumour tissue as extraction with Tyrode solution yields as much as extraction with trichloroacetic acid. Thus it appears that the tumour does not require the histamine for an internal function.

In the tumours other than the rat hepatoma, there is no relationship between histamine-forming capacity and growth ; growth continues without the need to produce histamine. This is particularly apparent with the hamster hepatoma where no enzyme activity is found at any time. It is also of interest that the foetal liver of the hamster possesses little histidine decarboxylase activity (Kameswaran and West, 1962). The other three tumours in the rat continued to grow when the enzyme activity was nearly zero, and further work is needed with other types of tumour.

### SUMMARY

1. There is a relationship between the histidine decarboxylase activity and growth of a transplantable rat hepatoma (F-hep). On the other hand, other rapidly-growing tumours of rat and human origin lack this enzyme.

2. The characteristics of the rat hepatoma resemble those of foetal rat liver and are unlike those of adult rat liver. For example, the enzyme forming histamine in the hepatoma, like that in the foetal liver, requires a pH value of 6·5 but no benzene.

3. The rat hepatoma induced in August rats has been transplanted through ten generations of cortisone-treated Wistar rats without loss of histidine decarboxylase activity. Regression of the tumour has always occurred in Wistar rats twenty days after grafting.

4. Potent inhibitors of histidine decarboxylase slightly reduce the growth and enzyme activity of the rat hepatoma.

It is a pleasure to acknowledge a grant from the British Empire Cancer Campaign.

### REFERENCES

Kahlson, G.—(1960) *Lancet*, i, 67.
Kameswaran, L. and West, G. B.—(1961) *J. Pharm., Lond.*, **13**, 191.—(1962) *J. Physiol.* **160**, 564.
Kato, L. and Gözsy, B.—(1956) *Int. J. Leprosy*, **24**, 447.
Mackay, D., Marshall, P. B. and Riley, J. F.—(1960) *J. Physiol.*, **153**, 31*P*.
Mishra, B. P. and Sanyal, R. K.—(1959) *J. Pharm., Lond.*, **11**, 127.
Northover, B. J.—(1958) *M. Pharm. Thesis*, Univ. of London.
Parratt, J. R. and West, G. B.—(1957) *J. Physiol.*, **137**, 179.
Price, S. A. P. and West, G. B.—(1960) *J. Pharm., Lond.*, **12**, 617.
Scott, K. G., Scheline, R. R. and Stone, R. S.—(1958) *Cancer Res.*, **18**, 927.
Telford, J. M. and West, G. B.—(1960) *J. Pharm., Lond.*, **12**, 254.—(1961*a*) *J. Physiol.*, **157**, 306.—(1961*b*) *J. Pharm., Lond.*, **13**, 75.
Toolan, H. W.—(1954) *Cancer Res.*, **14**, 660.
Waton, N. G.—(1956) *Brit. J. Pharmacol.*, **11**, 119.